# Supplementary material for: A Limited Course of Eculizumab in a Child with the Atypical Hemolytic Uremic Syndrome and Pre-B Acute Lymphoblastic Leukemia on Maintenance Therapy: Case Report and Literature Review
Source: J Clin Med. 2022 May 14;11(10):2779. doi: 10.3390/jcm11102779 (PMC9142928; doi:10.3390/jcm11102779)
Supplement: Supplementary file 1 [file jcm-11-02779-s001.zip › jcm-1647878-supplementary.pdf]

Supplementary material:

Supplementary diagram S1. Short diagnostic items and differentials of aHUS in patients with ALL

Supplementary Figure S1. Correlation of platelets with reticulocytes

Supplementary Table S1. Correlation matrix of variables used in aHUS monitoring

Supplementary diagram S1

1) Symptoms
    a. Common: anemia, fatigue, petechiae, oliguria, blood in urine (erythrocyturia),
    b. Uncommon: bloody diarrhea, fever
2) Laboratory tests for diagnosis and monitoring:
    a. Hemolysis, proteinuria
    b. elevated: urea, creatinine, LDH, free plasma hemoglobin
    c. reduced: thrombocytopenia, haptoglobin, C3
    d. normal: C4 (usually)
    e. ADAMTS 13 ≥ 10%
    f. Peripheral blood smear: schistocytes
3) Investigations towards etiology:
    a. Negative bacterial tests: E. Coli O157:H7, VTEC, verotoxin gene, Anti-VTEC lipopolysaccharide IgM, cultures not positive for other bacteria
    b. Negative viral tests: Hepatitis A, Hepatitis B, Hepatitis C, Hepatitis D, Cytomegalovirus, Herpes-simplex virus, Epstein-Barr virus
    c. Negative: systemic fungal infection
4) Other tests:
    a. Negative: Endomysial antibodies, ADNA, anti-neutrophil cytoplasm antibodies, warm and cold agglutinin antibodies, flow cytometry, methylmalonic aciduria, hyperhomocysteinemia
5) Renal histology: thrombotic microangiopathy
6) Bone marrow biopsy: less than 5% blasts in bone marrow
7) Lumbar puncture: $\leq 5 \times 10^6$/L WBC in cerebrospinal fluid

Supplementary Table S1. Correlation matrix of variables used in aHUS monitoring analyzed by Spearman rank correlation coefficient. p-values <0.01 are highlighted. Variables such as RPI/A *vs.* platelets, creatinine vs. LDH, and APRI *vs.* PLHBB show a strong positive correlation. In contrast, platelets *vs.* d-dimers, RPI/A *vs.* serum creatinine, and APRI *vs.* RPI/A show a strong negative correlation.

Variable	RPI/A	APRI	PLHBB	Platelets	D-dimers	LDH	Creatinine
RPI/A	1.00	-0.94	-0.83	0.93	-0.86	-0.87	-0.95
APRI	-0.94	1.00	0.90	-0.73	0.94	0.88	0.85
PLHBB	-0.83	0.90	1.00	-0.77	0.84	0.78	0.71
Platelets	0.93	-0.73	-0.77	1.00	-0.98	-0.80	-0.71
D-dimers	-0.86	0.94	0.84	-0.98	1.00	0.81	0.77
LDH	-0.87	0.88	0.78	-0.80	0.81	1.00	0.94
Creatinine	-0.95	0.85	0.71	-0.71	0.77	0.94	1.00

Legend: PLHBB - Plasma free hemoglobin

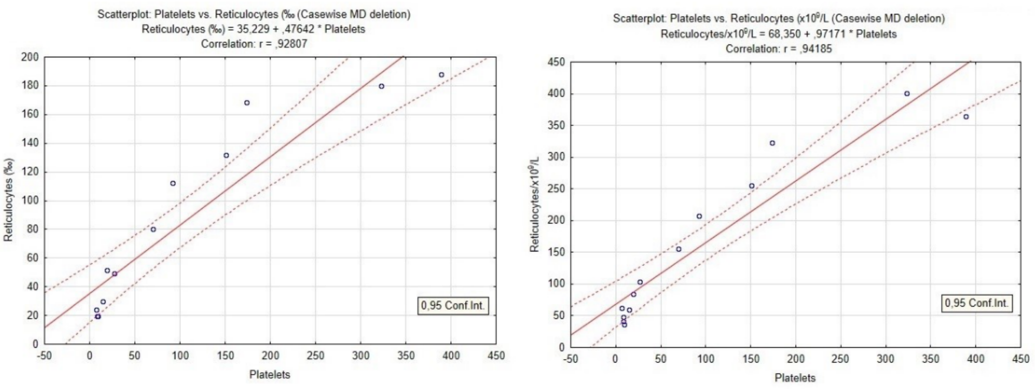

Supplementary Figure S1. Correlation of platelets with reticulocytes 10^9 (Spearman rank correlation coefficient $p<0.001$, $r=0.94$) and reticulocytes per million erythrocytes (Spearman rank correlation coefficient $p<0.001$, $r = 0.93$). Reticulocytes show a high degree of correlation with platelets but lower than RPI and RPI/A.
